# Dynamics of neutrophil phenotype and function in sickle cell disease

**DOI:** 10.3389/fimmu.2025.1591283

**Published:** 2025-05-02

**Authors:** Aafke E. Gaartman, Lydian A. de Ligt, Boukje M. Beuger, Anton T. J. Tool, Martijn Veldthuis, Taco W. Kuijpers, Rob van Zwieten, Bart J. Biemond, Robin van Bruggen, Erfan Nur

**Affiliations:** ^1^ Department of Hematology, Amsterdam University Medical Center, Amsterdam, Netherlands; ^2^ Red Cell Laboratory, Sanquin Research and Landsteiner Laboratory, Amsterdam, Netherlands; ^3^ Department of Pediatric Hematology, Emma Children’s Hospital, Amsterdam University Medical Center, Amsterdam, Netherlands; ^4^ Laboratory of Red Blood Cell Diagnostics and Iron, Sanquin Diagnostics, Amsterdam, Netherlands; ^5^ Department of Pediatric Immunology, Rheumatology and Infectious Diseases, Emma Children’s Hospital, Amsterdam University Medical Center, Amsterdam, Netherlands

**Keywords:** sickle cell disease, neutrophil activation, neutrophil adhesion, neutrohil aging, neutrophil phenotype, hemolysis, reactive oxygen species

## Abstract

**Introduction:**

While sickle cell disease (SCD) is primarily acknowledged as an erythrocyte disorder, emerging evidence suggests a role for altered neutrophil phenotype and function in SCD pathophysiology and disease severity. Given the conflicting findings in previous studies, we performed a comprehensive exploration of neutrophil characteristics in SCD patients during steady state and vaso-occlusive crisis (VOC), as well as in response to therapeutic interventions.

**Methods:**

Neutrophil phenotype was assessed by flow cytometry and functional properties were evaluated by measurement of neutrophil adhesion and reactive oxygen species (ROS) production.

**Results:**

A total of 49 SCD patients (of whom 19 during both steady state and VOC) along with 16 healthy ethnicity-matched and 30 non-matched controls, were included in the study. Differences were observed between neutrophils from patients compared to controls and between control groups. Neutrophil phenotype was more activated in SCD patients compared to non-matched controls. Neutrophil adhesion was increased in steady-state SCD patients compared to both ethnicity-matched and non-matched controls.

**Discussion:**

While neutrophil phenotype in SCD patients differed from non-matched controls, in contrast to earlier studies, the differences in neutrophil phenotype between SCD patients and ethnicity-matched controls were modest. *In vitro* neutrophil adhesion was higher in SCD patients than in ethnicity-matched and non-matched controls. Potential explanations for the discrepancies between earlier findings and our study are the large variation in neutrophil phenotypes between individuals, methodological variability between studies and differences in the time interval between blood sample collection and the measurements.

## 1 Introduction

SCD is a prevalent inherited hemoglobinopathy caused by a point mutation in the β-globin gene. The resulting hemoglobin S polymerizes upon deoxygenation, leading to the formation of the characteristic sickle erythrocytes (1). Clinically, these erythrocyte abnormalities result in hemolytic anemia and vaso-occlusion, causing periodic and painful episodes of VOC and ischemia-reperfusion injury in almost all organs.

**Figure 1:**
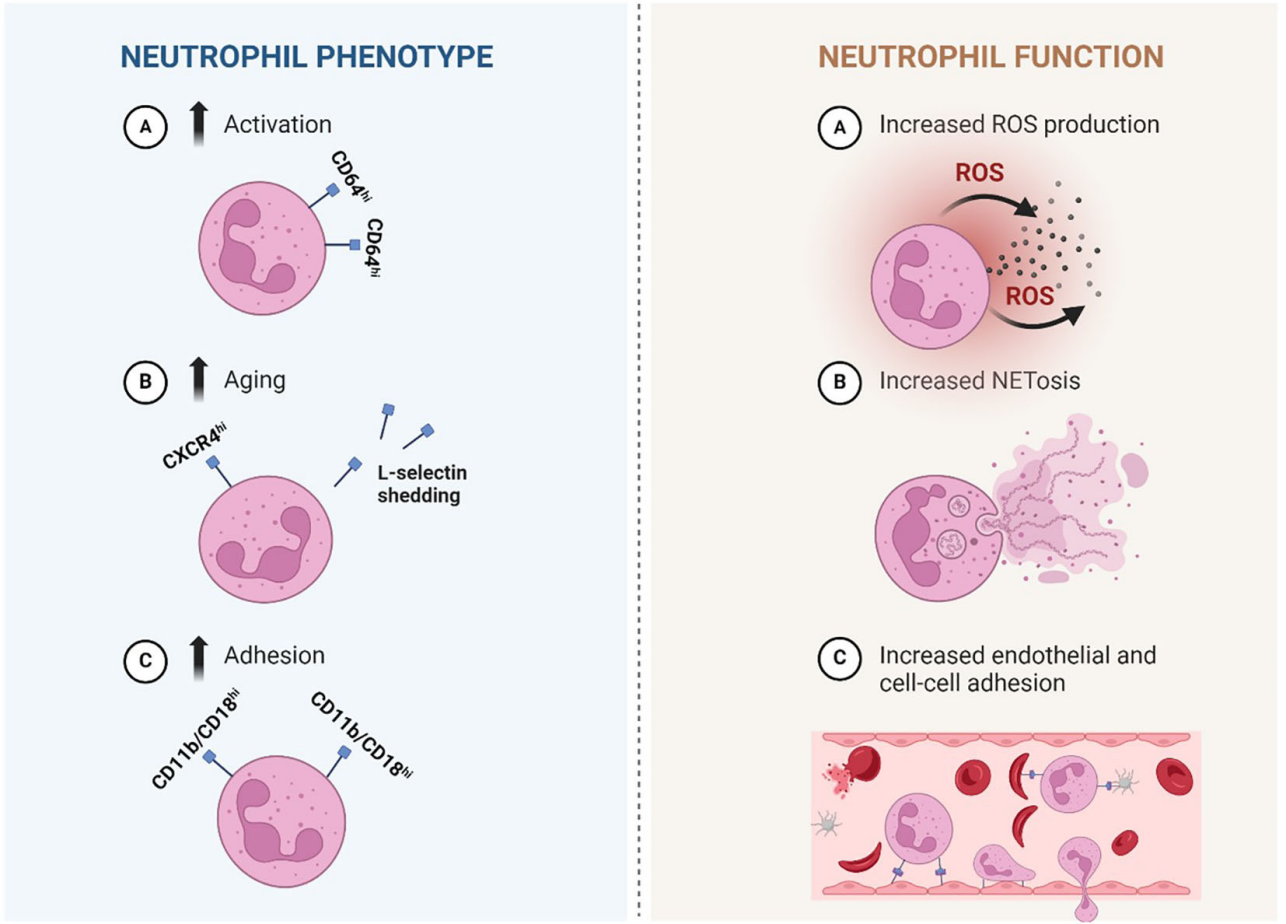
Simplified illustration of proposed neutrophil phenotypical and functional alterations in Sickle cell disease. Left: Neutrophil phenotype: Phenotypical changes that are described in sickle cell disease (SCD) are more activation, aging and adhesion. A: neutrophils that are activated express more CD64. B: increased aging is suggested by heightened expression of CXCR4 and reduced expression of L-selectin (CD62L) which is shed upon aging (and activation). C: increased expression of adhesion associated integrin CD11b/CD18 (α_m_β_2_) is described, suggesting a more adhesive neutrophil. Right: Neutrophil function: several changes in neutrophil function are described in SCD. A: Increased production of ROS, which are highly reactive chemicals used in the body’s response against pathogens. B: Increased production of NET-like structures made of DNA-histone complexes and proteins and can capture micro-organisms. A+B: both processes have also been linked to inflammation. C: Increased adhesion of neutrophils to the endothelium (followed by crawling over- and migration through the endothelium) and other cell types such as (sickled) erythrocytes and platelets, which can actively contribute to vaso-occlusion. SCD, sickle cell disease; CD, cluster of differentiation; ROS, reactive oxygen species; NET, neutrophil extracellular trap. Created in Biorender.

Although primarily considered an erythrocyte disorder, there is an increasing interest in other cell types contributing to the pathophysiology of SCD, such as neutrophils (2–5). Circulating neutrophils are suggested to play a pivotal role in the disease process (2, 3, 6, 7). Increased neutrophil counts are associated with clinical complications such as cerebral infarction, hemorrhagic stroke, acute chest syndrome (ACS) and early death (8–13). Additionally, neutrophils have been suggested to play a key role in initiating VOCs (14). Neutrophils interact with sickle erythrocytes and platelets via the neutrophil integrin α_m_β_2_ (CD11b/CD18) to form aggregates (15). Studies in sickle cell mice have shown that neutrophils adhere to vessel walls, decrease microvascular blood flow and interact with sickle erythrocytes, directly contributing to vaso-occlusion (16–19). Hemolysis and ischemia-reperfusion injury both lead to increased oxidative stress, resulting in chronic inflammation, increased neutrophil activation and adhesion (20). Neutrophil activation is characterized by increased expression of CD64 and decreased expression of CD62L (L-selectin) on neutrophils of SCD patients. In contrast, the neutrophil integrin α_m_β_2_ and CXCR4 expression are associated with increased adhesion and aging of neutrophils in SCD patients (21–23). Neutrophil functions affected in SCD include migration, phagocytosis, production of reactive oxygen species (ROS) and formation of neutrophil extracellular traps (NET), which collectively might contribute to disease severity in SCD (**Figure 1**) (19, 24–27).

Hydroxyurea remains one of the main therapeutic options for SCD and has a significant impact on neutrophil counts and phenotype (26, 28–31). Induction of hemoglobin F (HbF) is hydroxyurea’s primary mechanism of action, but the clinical efficacy could at least partly be attributed to its effect on neutrophils (32, 33). Hydroxyurea treatment reduces neutrophil counts, but its effects on neutrophil activation, adhesion and phenotype are still largely unknown. Hydroxyurea is thought to interfere with the dysregulated L-selectin shedding, increased ROS production and increased myeloperoxidase levels in SCD neutrophils (28, 32). However, signs of increased neutrophil activity, such as spontaneous degranulation and enhanced production of NETs, have shown to persist in hydroxyurea-treated SCD patients (26).

This study aims to further investigate neutrophil phenotype and activity in SCD patients during steady state and VOC, as well as elucidate the effects of treatment, *e.g.* hydroxyurea or allogeneic hematopoietic stem cell transplantation (HSCT), on neutrophil characteristics.

## 2 Methods

### 2.1 Study population

In this prospective study, blood was collected from adult patients with a confirmed diagnosis of sickle cell anemia (HbSS) and heterozygous HbSβ^0^-thalassemia (HbSβ^0^-thal) during steady state and hospital admission for VOC. To assess the effects of treatment on neutrophil phenotype and functionality, we included a subgroup of patients before and during hydroxyurea treatment and before and after HSCT, which they received as standard of care. Healthy ethnicity-matched individuals were included as a control group. Controls were considered matched if at least one parent originated from an SCD-endemic region. Additionally, a healthy non-matched control group, primarily of Caucasian origin, was included as a quality control in all experiments. Results of SCD patients are compared with both ethnicity-matched healthy controls and non-ethnicity-matched healthy controls, hereafter referred to as ‘matched controls’ and ‘non-matched controls’.

Potential participants with a history of immune-related disorders or current use of medication influencing immune cells or inflammation at the moment of blood collection were excluded. Blood transfusion within 3 months were additional exclusion criteria. Participants who received a blood transfusion more than 3 months before inclusion, who still had an HbA1 of >10%, were also excluded from final analyses.

This study was approved by the Institutional Review Board of Amsterdam UMC and performed in accordance with the Declaration of Helsinki 2013. Informed consent was obtained from all participants before study inclusion.

### 2.2 Study procedures

Blood was obtained from SCD patients during a visit to the outpatient clinic or within 48 hours after hospital admission for VOC. In patients starting hydroxyurea, a second blood sample was collected after treatment for >90 days at a stable dose. In patients undergoing HSCT, a second blood sample was drawn after successful engraftment and at least mixed chimerism (≥6 months after transplantation). Patients that were included during VOC had a follow-up steady-state blood sample collected at least 4 weeks after the VOC.

Standard complete blood counts were performed in EDTA-anticoagulated blood and markers of hemolysis (levels of bilirubin and lactate dehydrogenase and reticulocyte counts) were measured in heparinized plasma with spectrophotometry according to local protocols. Erythrocyte assays were conducted within 24 hours after venous blood was collected in EDTA-anticoagulated tubes and stored at 4°C. Neutrophil assays were performed within 24 hours of collection in EDTA-anticoagulated blood samples, stored at room temperature.

### 2.3 Study assays

To assess neutrophil phenotype, neutrophil surface antigen expression was measured by flow cytometry. A set of directly conjugated antibodies was used. To facilitate readability, markers were roughly divided into subgroups of activation, adhesion and maturation (**Supplementary Table S1**). Flow cytometry data were quantified using a Canto II flow cytometer (BD Biosciences) and analyzed with FacsDiva software (Version 9). For FITC/AF488, the instance laser lines were 488 nm with filter set long-pass (LP) 502 nm and band-pass (BP) 530/30 nm. For APC/AF647, the instance laser lines were 633 nm with filter set no LP and BP 660/20 nm. Erythrocytes in whole blood were lysed twice with ice-cold lysis buffer (4.15 g NH_4_Cl, 0.5 g KHCO_3_ and 18 mg EDTA in 500ml H_2_O). Antibodies were added to the remaining cells. To distinguish neutrophils from other white blood cells, neutrophils were gated based on forward and side scatter. Results were depicted as Mean Fluorescent Intensity (MFI).

For *in vitro* neutrophil adhesion and neutrophil ROS production measurements, neutrophils were isolated from whole blood using a Percoll gradient and lysis buffer as previously described (34, 35). Neutrophils were kept at room temperature in a HEPES-buffered saline solution (20 mM HEPES, 132 mM NaCl, 6.0 mM KCl, 1.0 mM CaCl_2_, 1.0 mM MgSO_4_, 1.2 mM potassium phosphate, 5.5 mM glucose and 0.5% (w/v) human serum albumin, pH 7.4). For the adhesion assay, neutrophils (5 x 10^6^/ml) were incubated with calcein-AM (1 μM; Molecular Probes) for 30 minutes at 37°C, washed twice, and resuspended in HEPES medium at a concentration of 2 x 10^6^/ml. Adhesion was determined in an uncoated 96-well MaxiSorp plate (Nunc, Wiesbaden, Germany). Calcein-labeled cells (1.6 x 10^6^/ml) were stimulated with 20 ng/ml granulocyte colony-stimulating factor (G-CSF), 10 mM dithiothreitol (DTT; Sigma Aldrich, St. Louis, MO, USA), 20 μg/ml Pam3Cys (EMC Microcollections, Tübingen, Germany), 20 ng/ml bacterial Toll Like Receptor-4 ligand lipopolysaccharide (LPS; isolated from *E. coli* strain 055:B5, Sigma Aldrich) in the presence of 50 ng/ml lipopolysaccharide-binding protein (LBP; R&D Systems, Minneapolis, MN, USA), 1 μM platelet-activation factor (PAF; Sigma Aldrich), 1 μM N-formyl-Met-Leu-Phe (fMLP), 10 ng/ml tumor necrosis factor-α (TNFα) or 100 ng/ml phorbol myristate acetate (PMA). Plates were incubated for 30 minutes at 37°C and washed with phosphate-buffered saline (PBS) twice. Adherent cells were lysed in 0.5% (w/v) Triton X-100 in PBS for 5 minutes at room temperature. Fluorescence was assessed using an Infinite F200-pro plate reader (Tecan, Mannedorf, Switzerland) at an excitation wavelength of 485 nm and an emission wavelength of 535 nm. Adhesion was determined as the percentage of the total input of calcein-labeled cells.

The production of reactive oxygen species was measured with an Amplex Red kit (Molecular Probes, Eugene, OR, USA). In short, neutrophils (0.25 x 10^6^/ml) were stimulated with 1 mg/ml unopsonized zymosan (MP Biomedicals, Solon, OH, USA), serum-treated zymosan (STZ), PMA (100 ng/ml; Sigma-Aldrich), fMLP (1 µM; Sigma-Aldrich) or PAF/fMLP (1/1 µM; Sigma-Aldrich) in the presence of Amplex Red (25 μM) and horseradish peroxidase (0.5 U/ml). Fluorescence was measured at 30-second intervals for 30 minutes with an Infinite F200-pro plate reader at an excitation wavelength of 535 nm and an emission wavelength of 595 nm. The activity of the NADPH oxidase of neutrophils was determined as nmol H_2_O_2_/minute x 10^6^ cells. The maximal slope of hydrogen peroxide release was measured at a 2-minute interval.

### 2.4 Erythrocyte deformability

Erythrocyte deformability was assessed under shear stress and decreasing oxygen concentrations. Erythrocytes were counted using an ADVIA 2120 hematology cell counter (Siemens, Munich, Germany) and diluted according to standard procedure to a fixed cell number in oxy iso fluid before the cell suspension was introduced in a laser optical rotational red cell analyzer (Lorrca, RR Mechatronics, Zwaag, The Netherlands), with oxygenscan module, as described previously (36). The deformability of erythrocytes in relation to deoxygenation is interpreted as a maximal and minimal elongations index (EImax and EImin, defined as the elongation index at normal (47 mmHg) or low oxygen pressure (10 mmHg), respectively, and the point of sickling (PoS), defined as the oxygen tension at which 5% reduction of the EImax is observed.

### 2.5 Statistics

Data were analyzed using SPSS version 26 (IBM, Armonk, NY, USA) and GraphPad Prism version 9 (GraphPad Software, Boston, MA, USA). Data are presented as mean ± standard error of the mean (SE) or standard deviation (SD), or median and interquartile range (IQR) depending on the distribution of data. Patient characteristics were compared between the groups using standard descriptive statistics. *Post-hoc* analyses with Bonferroni correction or Dunns rule for multiple testing were performed when more than 2 groups were compared. Paired data were analyzed using the paired t-test, Wilcoxon signed-rank test or McNemar test as appropriate.

## 3 Results

### 3.1 Baseline characteristics

A total of 49 SCD patients (median age 25.0 years [IQR 20.0-32.5], 41% female; **Table 1**), 16 matched controls (29.0 years [27.0-43.5], 50% female) and 30 non-matched controls (28.0 years [26.0-31.0]) were included in the final analyses. Nineteen patients were included during VOC and in steady state. Nine of the 48 steady-state patients were included before initiation of hydroxyurea with a follow-up sample after hydroxyurea treatment at a stable dose for >90 days. Seven of the 48 patients were included before HSCT with a follow-up sample ≥6 months after transplantation.

**Table 1:** Baseline characteristics of patients with sickle cell disease.

Baseline characteristics	SCD patients (N=49)^1^
**Age, years** ^*^	25.0 [20.0-32.5]
**Female sex at birth^+^**	20 (41%)
Genotype	
HbSS	44 (90%)
HbSβ^0^	5 (10%)
Laboratory parameter	
Hemoglobin (mmol/L)	5.3 (4.8-6.0)
Platelets (10^9^/L)	348 (304-414)
Total leukocyte count (10^9^/L)	9.4 (7.9-10.9)
Absolute neutrophil count (10^9^/L)	4.8 (3.8-6.3)
Absolute lymphocyte count (10^9^/L)	3.1 (2.2-3.8)
Absolute monocyte count (10^9^/L)	0.9 (0.5-1.1)
Total bilirubin (umol/L)	50 (31-95)
Lactate dehydrogenase (IU/L)	414 (341-530)
Absolute reticulocyte count (10^9^/L)	268 (215-328)
Medical history	
Hydroxyurea use at baseline^*^	14 (29%)
Frequent VOCs (>1/year)	21 (44%)
ACS	21 (44%)
CVA	4 (8%)
Cholelithiasis	27 (56%)
Microalbuminuria	17 (35%)
History of chronic ulcera	5 (10%)
Pulmonary hypertension	2 (4%)
Retinopathy	7 (15%)
Osteonecrosis	6 (13%)
Priapism	4 of 29 men (14%)

Data are presented as medians with interquartile ranges or absolute numbers with percentages. ^1^Of one patient, no baseline sample was obtained. Therefore, a total of 49 patients were included, of whom 48 in steady state. *Use of hydroxyurea was assessed based on anamnestic evaluation, drug prescriptions, and effects on mean corpuscular volume and fetal hemoglobin values.

SCD, sickle cell disease; VOC, vaso-occlusive crisis; ACS, acute chest syndrome; CVA, cerebrovascular accident.

### 3.2 Steady-state SCD patients versus healthy controls

Results of neutrophil antigen expression in steady-state SCD patients and controls are depicted in **Table 2**. Expression of CD62L (L-selectin), which is shed upon activation, was significantly lower in steady-state SCD patients than in matched controls (median MFI: 2229 versus 3947, *p*=0.006). No other differences were observed between steady-state SCD patients and matched controls. CXCR4 expression was comparable between SCD patients and matched controls, but significantly higher in non-matched controls (*p*=0.019). CD64 expression was comparable between SCD patients and matched controls, but significantly lower in non-matched controls (*p*<0.0001). Neutrophil expression of the α_m_β_2_ integrin CD11b/CD18 did not differ between SCD patients and the two control groups.

**Table 2:** Neutrophil antigen expression in steady-state SCD patients and healthy controls.

Antigen expression – MFI	Steady state SCD N=48	Matched controls N=16	Non-matched controls N=30	p*
Activation markers				
CD177	13501 [7830-19060]	18330 [11999-26713]	11081 [6364-23484]	.126
CD62L	2229 [1201-3248]	3947 [2559-5349]	3166 [1632-5444]	**.006^1^**
CXCR4	385 [320-748]	393 [273-686]	643 [460-843]	**.019^2^**
CD32	14499 [12568-16613]	14396 [12562-16287]	14690 [12983-16244]	.999
CD64	797 [444-1512]	1158 [318-2356]	237 [118-349]	**<.0001^3^**
CD66b	3390 [1715-6972]	4465 [2825-7303]	4408 [3203-5539]	.478
CD63	1456 [1041-2333]	1227 [651-1482]	1018 [727-1711]	.065
7D5	9148 [5132-14215]	10162 [3887-13557]	11504 [6020-15664]	.657
CD55	637 [289-1361]	555 [367-1800]	641 [458-1036]	.969
CD59	23407 [20225-28010]	22948 [16100-25782]	22616 [20348-27578]	.572
CD163	32 [0-157]	28 [12-54]	17 [0-68]	.699
Adhesion markers				
CD11b	8971 [7810-14300]	10994 [7741-15654]	8521 [7843-10538]	.219
CD18	10581 [8374-12961]	10535 [8260-13033]	10972 [9079-13771]	.571
Maturation markers				
EMR3	5688 [4853-6611]	5619 [4641-6510]	5930 [5289-7176]	.436
CD16	58604 [50745-75741]	51396 [43176-71214]	75638 [49735-82653]	.113

Neutrophil antigen expression at baseline in steady state sickle cell disease patients compared to matched and non-matched controls. Neutrophil markers used for phenotyping of neutrophils were generally subdivided in groups of activation, adhesion and maturation based on literature to facilitate readability.

*The *p* value represents the significance of the comparison between the three groups unless stated otherwise, a specification for significant *p* values is provided:

^1^Significant difference between matched controls and steady-state SCD patients.

^2^Significant difference between non-matched controls and steady-state SCD patients.

^3^Significant difference between non-matched and matched controls, and between non-matched controls and steady-state SCD patients.

All analyses were performed with correction for multiple testing with Dunn’s test.

SCD, sickle cell disease; MFI, mean fluorescent intensity; CD, cluster of differentiation; CXCR, chemokine receptor; EMR3, EGF-like module-containing mucin-like hormone receptor-like 3.

The bold values represent statistical significance.

No significant correlations between markers of hemolysis (levels of bilirubin and lactate dehydrogenase and reticulocyte counts), a proposed driver of neutrophil activation, and expression of markers of neutrophil activation were found (data not shown). We hypothesized that hemolysis would be a driver of neutrophil activation due to the release of reactive oxygen species during hemolysis, however this was not supported by our data. We believe this could be due to the variation in neutrophil data and limited sample size.

**Figure 2:**
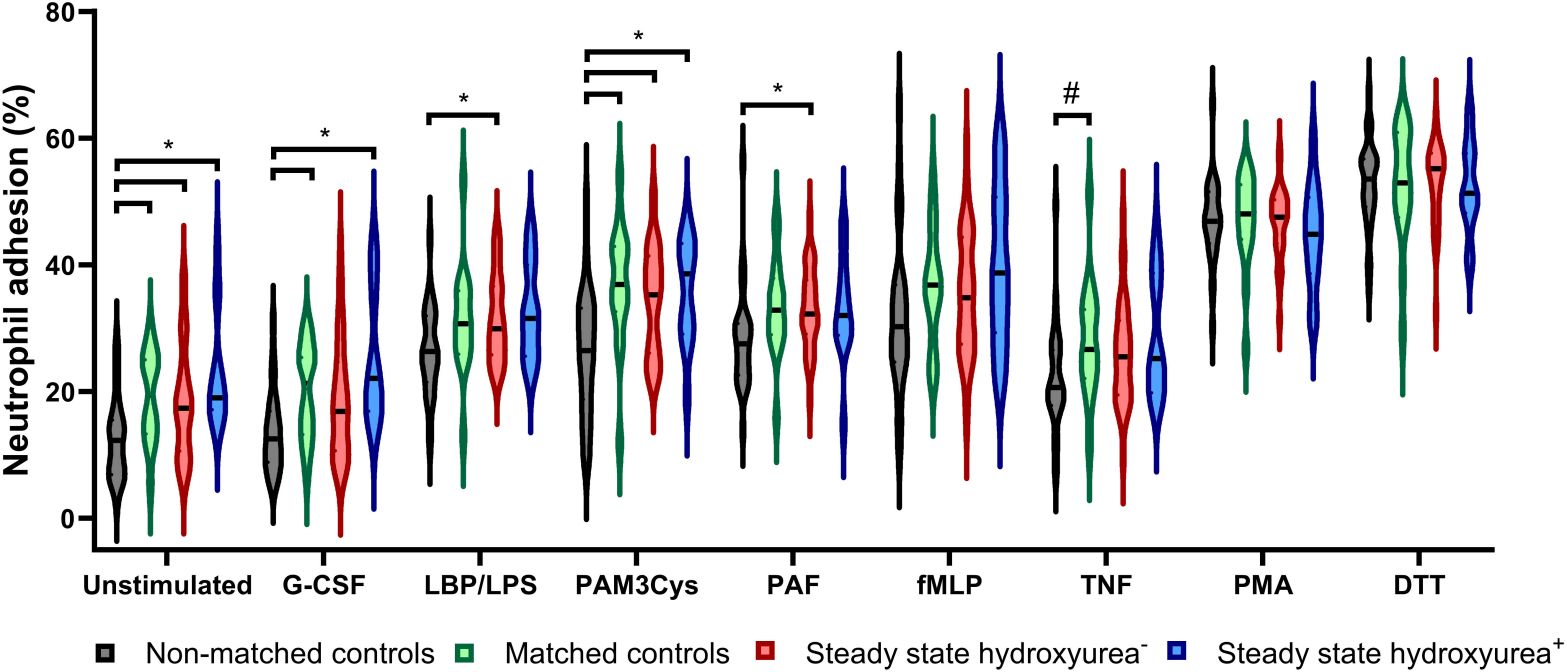
Neutrophil adhesion in steady-state SCD patients compared to controls. Results of neutrophil adhesion assay in healthy matched and non-matched controls and steady-state sickle cell disease patients with, or without hydroxyurea treatment. Results are expressed as percentage (%) of total input of calcein-labeled cells. Grey, non-matched controls (*n*=30); green, matched controls (*n*=16); red, unpaired steady-state SCD without hydroxyurea (*n*=34); blue, unpaired steady-state SCD patients during hydroxyurea treatment (*n*=14). Unstimulated, non-matched controls significantly lower compared to all other groups; G-CSF, non-matched significantly lower than matched controls and hydroxyurea^+^; LBP/LPS, non-matched controls significantly lower than hydroxyurea^-^; Pam3Cys, non-matched controls significantly lower compared to all other groups; PAF, non-matched controls significantly lower than hydroxyurea^-^; TNF, significant difference between non-matched controls and matched controls. *Significant after correcting for multiple testing. #Not significant after correcting for multiple testing. Created in Biorender.

Neutrophil adhesion was not different between SCD patients and matched controls. However, neutrophil adhesion was significantly lower in non-matched controls as compared to SCD patients and matched controls, both under unstimulated and stimulated conditions (**Figure 2**; **Supplementary Table S2**). ROS production of neutrophils stimulated by zymosan was lower in SCD patients compared to matched controls (median 0.40 [0.36-0.43] versus 0.44 nmol H_2_O_2_/minute x 10^6^ cells [0.40-0.50], *p*=0.029). There were no differences in unstimulated ROS production between SCD patients and matched and non-matched controls (**Supplementary Table S2**).

No differences in neutrophil antigen expression were seen between patients using and those not using hydroxyurea. Neutrophil adhesion under unstimulated conditions and upon G-CSF stimulation seemed higher in patients using hydroxyurea than in those not using hydroxyurea, although this did not reach statistical significance (**Figure 2**). No differences in ROS production were observed in SCD patients using and those not using hydroxyurea (data not shown).

### 3.3 Steady state versus vaso-occlusion

In the VOC subgroup (*n*=19), neutrophil expression of CXCR4 was significantly higher in steady state than during VOC with paired data analysis (median MFI: 500 versus 265, *p*=0.015), suggesting a younger subset of neutrophils during VOC. Likewise, expression of CD11b was significantly higher in steady state compared to VOC (median MFI: 9117 versus 6875, *p*=0.041). There was no difference in CD18 expression (**Table 3**).

**Table 3:** Neutrophil antigen expression in SCD patients in steady state and during VOC – paired data.

Antigen expression – MFI	Steady state N=18	VOC N=19	*p*
Activation markers			
CD177	15934 [10946-20513]	16170 [9606-24068]	.394
CD62L	1926 [1186-2262]	3210 [1676-5271]	.156
CXCR4	500 [332-827]	265 [146-533]	**.015**
CD32	13401 [11106-16863]	14469 [12526-16856]	.496
CD64	478 [314-732]	710 [365-773]	.140
CD66b	5822 [2455-8981]	4250 [2797-10677]	.776
CD63	1333 [1052-2180]	933 [662-1716]	.211
7D5	13624 [6362-14272]	10240 [8025-14964]	.733
CD55	471 [145-818]	292 [140-753]	.363
CD59	21737 [17684-23935]	24228 [19562-27873]	.334
CD163	45 [0-151]	18 [0-181]	.875
Adhesion markers			
CD11b	9117 [8170-14651]	6875 [5920-10780]	**.041**
CD18	10148 [7421-12077]	11568 [9169-13209]	.650
Maturation markers			
EMR3	5705 [4708-6586]	5088 [4461-5916]	.140
CD16	54533 [43314-71090]	46507 [39429-64722]	.211

Neutrophil antigen expression in steady state versus during VOC. Neutrophil markers were generally subdivided in groups of activation, adhesion and maturation based on literature to facilitate readability.

VOC, vaso-occlusive crisis; MFI, mean fluorescent intensity; CD, cluster of differentiation; CXCR, chemokine receptor; EMR3, EGF-like module-containing mucin-like hormone receptor-like 3.

Of one patient no paired steady-state data was available.

The bold values indicate statistical significance.

Neutrophil adhesion upon stimulation with LBP/LPS was significantly lower in steady state than during VOC (median 29.5% [24.7-40.3] versus 34.1% [25.7-46.1], *p*=0.043). Neutrophil adhesion upon stimulation with other stimuli did not differ between VOC and steady state. There were no differences in neutrophil ROS production between steady state and VOC (**Supplementary Table S3**).

### 3.4 Hydroxyurea treatment and hematopoietic stem cell transplantation

Paired data of neutrophil phenotypical changes in a subgroup of patients starting hydroxyurea treatment or undergoing HSCT are depicted in **Table 4**. Upon hydroxyurea treatment (n=9), a decrease in the expression of the activation marker CD63 was observed (from median MFI 1939 to 1298, *p*=0.038). CD62L seemed to increase following the use of hydroxyurea, but this increase was not statistically significant (median MFI 2644 to 3654, p=0.441).

**Table 4:** Neutrophil antigen expression in SCD patients treated with hydroxyurea and hematopoietic stem cell transplantation – paired data.

Antigen expression – MFI	Before HU N=9	During HU N=9	*p*	Before HSCT N=7	After HSCT N=7	*p*
Activation markers						
CD177	8639 [6238-20042]	19702 [14915-24535]	.139	14707 [2536-18865]	9614 [7934-12832]	.753
CD62L	2644 [2183-4012]	3654 [2364-5642]	.441	1291 [914-4147]	4159 [2913-5054]	.345
CXCR4	399 [304-911]	531 [500-677]	.889	531 [370-893]	839 [550-2113]	**.046**
CD32	14852 [14015-15939]	17419 [14420-18848]	.374	14380 [11272-21121]	13083 [5540-14432]	.249
CD64	662 [272-1768]	676 [296-1265]	.859	980 [674-1639]	222 [0-567]	**.028**
CD66b	2385 [1431-5092]	4338 [1590-9412]	.214	5632 [2534-8015]	6217 [1128-11943]	.463
CD63	1939 [1285-2735]	1298 [817-1889]	**.038**	1872 [1501-2825]	4699 [2858-9602]	**.028**
7D5	10844 [3637-14730]	8516 [4898-10760]	.173	8374 [5224-18095]	11092 [8354-15832]	.600
CD55	1540 [426-2514]	722 [480-1365]	.139	591 [253-1142]	3042 [1595-3649]	**.028**
CD59	25931 [20318-31239]	22122 [18475-29622]	.594	25541 [19978-32079]	18314 [17097-21936]	.249
CD163	56 [10-249]	51 [0-140]	1.000	58 [0-151]	399 [26-636]	**.028**
Adhesion markers						
CD11b	11473 [8426-18147]	8689 [7434-11912]	.086	8079 [5288-11805]	9946 [7396-14495]	.249
CD18	11209 [8774-15221]	9990 [9085-11732]	.374	9940 [8650-14857]	8527 [4342-11153]	.116
Maturation markers						
EMR3	5332 [4661-6585]	5094 [4210-5927]	.051	6059 [4443-7506]	2989 [1182-4744]	**.028**
CD16	66497 [55999-84963]	61606 [45846-78799]	.374	70608 [50677-110605]	39603 [15322-60529]	**.028**

Neutrophil antigen expression in patients before and during the use of hydroxyurea, and before and after hematopoietic stem cell transplantation (HSCT). Neutrophil markers used for phenotyping of neutrophils were generally subdivided in groups of activation, adhesion and maturation based on literature to facilitate readability.

SCD, sickle cell disease; MFI, mean fluorescent intensity; HU, hydroxyurea; HSCT, hematopoietic stem cell transplantation; CD, cluster of differentiation; CXCR, chemokine receptor; ICAM, intercellular adhesion molecule; VCAM, vascular cell adhesion molecule; EMR3, EGF-like module-containing mucin-like hormone receptor-like 3.

The bold values indicate statistical significance.

Functional neutrophil assays of the hydroxyurea subgroup are shown in **Supplementary Table S4**. Unstimulated adhesion of neutrophils was significantly higher during hydroxyurea use than before starting hydroxyurea (from median 14.3% before [IQR 9.4-17.9] to 18.8% after [14.5-24.1], *p*=0.038). Trends were seen towards higher LBP/LPS, Pam3Cys and TNF-a stimulated neutrophil adhesion upon hydroxyurea treatment, although these differences were not statistically significant. Under hydroxyurea treatment, the neutrophil oxidative burst stimulated by PAF/fMLP significantly decreased (1.32 [1.19-1.49] to 1.14 nmol H_2_O_2_/minute x 10^6^ cells [0.97-1.23], *p*=0.036).

After HSCT (*n*=7), neutrophil expression of CXCR4 increased (median MFI 531 to 839, *p*=0.046; **Table 4**). Expression of CD62L also seemed to increase after HSCT, though the difference was not statistically significant. Expression of CD64 decreased (from median MFI 980 to 222, *p*=0.028), while the complement related protein CD55 increased after HSCT (from median MFI 591 to 3042, *p*=0.028). The expression of EMR3 and CD16 decreased after HSCT (from median MFI 6059 to 2989, *p*=0.028, and 70608 to 39603, *p*=0.028, respectively). There were no differences in neutrophil adhesion or ROS production measurements (**Supplementary Table S5**).

### 3.5 Point of sickling

Point of sickling did not differ between steady state and VOC (20.9 mmHg [17.3-23.6] versus 21.8 mmHg [19.9-23.8], *p*=0.38). During treatment with hydroxyurea, the PoS was lower as compared to baseline in the patients measured both before and after starting hydroxyurea (*n*=9), though this did not reach statistical significance (from 24.5 before [14.7-25.5] to 15.2 mmHg after [15.0-19.9], *p*=0.091). The PoS was no longer detectable after transplant, comparable to healthy controls (data not shown). No significant changes in EImax and EImin were observed between steady state and VOC or after hydroxyurea (data not shown).

## 4 Discussion

While neutrophil involvement in SCD pathophysiology is widely described, the *ex vivo* assessment of SCD neutrophil phenotype and function has yielded conflicting results (37). Our study extensively evaluated these aspects in distinct SCD patient subgroups, comparing them with both (ethnicity-) matched and non-matched controls. Neutrophil characteristics were largely comparable between SCD patients and matched controls, with some differences observed when SCD patients were compared with non-matched controls. Treatment effects on neutrophil characteristics were observed, though most previously published findings could not be confirmed.

Neutrophils of SCD patients have previously been characterized as activated and aged, marked by reduced L-selectin and increased CD64 and CXCR4 expression (3, 21, 23, 28, 38). While reduced L-selectin expression in SCD patients suggested increased neutrophil activation in our study, other markers such as CXCR4 and CD64 did not. Additionally, while some previous studies have reported increased expression of adhesion-associated integrin CD11b/CD18 on SCD neutrophils, we and others did not observe increased CD11b/CD18 expression (3, 21, 22, 28, 39, 40).

Variations in experimental methods, including centrifugation, processing time, and exposure to activating compounds such as lysis buffer during neutrophil isolation, have been proposed as potential contributors to discrepancies in antigen expression across studies (22). Furthermore, sample handling practices vary. In some studies, neutrophils were fixed on ice immediately after collection, in other studies samples were stored at room temperature until isolation (22, 28). In the present study, experiments were performed within 24 hours after collection of blood and not directly due to practical constraints, which potentially influenced the results.

Notably, differences were observed between neutrophils of matched and non-matched controls, raising the question of whether disparities in neutrophil activity between SCD patients and healthy controls may be, in part, related to ethnicity. Total leukocyte, neutrophil, and platelet counts are lower in African Americans compared to Caucasians and increased platelet-erythrocyte and platelet-monocyte aggregates are described in African Americans compared to Caucasians (41–44). Additionally, neutrophils of African Americans showed an upregulation of CD11b/CD18 at a lower dose of IL-8 stimulation than neutrophils of Caucasians (22). Our CD11b/CD18-dependent functional adhesion assay revealed significant differences between SCD patients and non-matched controls, but not between SCD patients and matched controls. This might support the notion that both neutrophil numbers, as well as phenotypical and functional characteristics, may differ across ethnicities. Some studies into neutrophils in SCD patients lack an ethnicity-matched control group, which might contribute to data variation. Based on the differences observed between the non-matched and ethnicity-matched control groups, we recommend including ethnicity matched healthy controls for future studies on neutrophil function and phenotype.

In the present study, we did not observe any differences in ROS production at baseline (unstimulated) or after stimulation between SCD patients in steady state and during VOC and both control groups. Benkerrou et al. reported increased ROS production of SCD neutrophils upon stimulation, while Evans et al. observed an impaired (decreased) oxidative burst capacity under stimulated conditions (28, 45). As Benkerrou et al. included pediatric SCD patients, this could contribute to the differences in results between the studies. In addition, differences in the methods used to measure ROS production, as well as differences in handling of the samples could also account for the contradictory results in these studies (28).

Differences in neutrophil adhesion could be restricted to VOCs (40, 46). Although our study found no statistically significant differences during VOC compared to steady-state conditions or matched controls, the observed trends might suggest biologically meaningful changes. In addition, the LBP/LPS-stimulated condition revealed increased adhesion in SCD neutrophils during VOC. In contrast, CD11b expression was decreased during VOC compared to steady-state, possibly reflecting shifts in intravascular neutrophil subpopulations, where more activated and adhesive neutrophils may have adhered and transmigrated *in vivo*. Alternatively, stress-induced neutrophilia during VOC (indeed, the total leukocyte count and absolute neutrophil count increased during VOC in our study population) may result in the presence of a more immature, less activated, and less adhesive neutrophil population in the circulation, explaining the decreased expression of the aging marker CXCR4.

Hydroxyurea treatment is hypothesized to exert direct and indirect effects on neutrophils, potentially normalizing activation markers and ROS production, although conflicting results exist (3, 26, 28). In our study, a decrease in CD63 expression, indicative of azurophilic granule release, was found after hydroxyurea treatment, suggesting a deactivating effect. The trend towards increased L-selectin expression post-treatment also suggests de-activation. Other activation markers such as CD64 and CXCR4 as well as adhesion molecules CD11b and CD18 remained unchanged, although it is difficult to compare these markers due to different mechanisms that lead to their expression. Surprisingly, the baseline neutrophil adhesion (a CD11b/CD18 dependent assay) increased upon hydroxyurea treatment, despite unaltered expression of CD11b/CD18. Our study does not provide conclusive information on the cause of this observation and this might be influenced by the small number of patients in the hydroxyurea group and large assay variation. One hypothesis could be that changes in the composition of intravascular neutrophils occur, as hydroxyurea leads to reduced generation of neutrophils that might adhere less readily to the endothelial cells *in vivo*. In this study, we measured surface expression of CD11b/CD18 and not changes in its activity that can occur through functional upregulation by conformational changes (inside-out signaling) (38, 47). Integrin activation is regulated on many different levels, and it might be that hydroxyurea treatment influences integrin clustering or conformation by other means (48). ROS production upon stimulation with PMA and PAF/fMLP was reduced upon hydroxyurea treatment, falling below levels observed in healthy controls. This observation is in line with previous findings by Benkerrou et al. (28), although the mechanism(s) behind this lower activity remain unclear. When comparing all steady state SCD patients using hydroxyurea to those without hydroxyurea treatment, a trend towards increased expression of CD64 and increased unstimulated adhesion in patients using hydroxyurea was observed. This suggests an association between hydroxyurea use and increased neutrophil adhesion, possibly due to indication bias, as patients with more severe disease are more likely to be prescribed hydroxyurea.

All seven transplanted patients included in the study showed successful engraftment; their erythrocyte phenotypes were comparable to those of their donors with normalized PoS levels. A decrease in neutrophil CD64 expression, together with a non-significant rise in L-selectin expression after transplantation and decreased CD55 expression, suggests reduced neutrophil activation. However, conflicting results were observed for markers such as CXCR4, CD163, and CD63 after transplantation, indicating incomplete normalization of neutrophil phenotype despite a complete normalization of the PoS. This disparity may be explained by the presence of sickle cell trait in 57% of donors and post-transplant medication like sirolimus in all patients. Lastly, it is important to note that the comparison between pre- and post-HSCT antigen expression is not strictly paired, as pre-HSCT neutrophils are patient-derived, where post-HSCT neutrophils are donor-derived.

The strength of our study lies in providing an extensive evaluation of neutrophil characteristics in SCD patients, featuring paired data during VOC and from those undergoing treatment with hydroxyurea or HSCT. However, limitations include the relatively small sample sizes of the VOC, hydroxyurea, and HSCT subgroups, reducing statistical power. Considerable variability in antigen expression and functional assays further challenge robust conclusions within these groups. Differences in experimental methods complicate comparisons with other studies. Addressing limitations in experimental methods, such as delays in measurements after collecting blood samples, storing temperature and the use of lysis buffer, is essential.

In conclusion, our study highlights the significant influence of ethnicity on neutrophil phenotype, which may have biased previous observations. While confirming some findings from previous reports regarding neutrophil characteristics in SCD, our study emphasizes the substantial variation in study outcomes. Larger studies with enhanced statistical power and uniformed protocols are needed to comprehensively unravel the function and phenotype of neutrophils in SCD.

## Data Availability

The raw data supporting the conclusions of this article will be made available by the authors, without undue reservation.
