# Supplementary material for: Dynamics of neutrophil phenotype and function in sickle cell disease
Source: Front Immunol. 2025 May 2;16:1591283. doi: 10.3389/fimmu.2025.1591283 (PMC12081264; doi:10.3389/fimmu.2025.1591283)
Supplement: Supplementary file 2 [file Table1.docx]

**Supplementary Table 1. Antibody overview, for neutrophil surface antigen expression.**

| **Antibody list** | **Label** | **Manufacturer** | **Productnumber/clone** |
| --- | --- | --- | --- |
| **Activation markers** | | | |
| CD177 | FITC | BioRad | MCA2045F/MEM-166 |
| CD62L | APC | BD Pharmingen | 559772/DREG-56 |
| CXCR4 | AF488 | R&D Systems | FAB173G/44717 |
| CD32 | FITC | BioRad | MCA1075F/AT10 |
| CD64 | FITC | BioRad | MCA756F/10.1 |
| CD66b | FITC | BioRad | MCA216F/80H3 |
| CD63 | AF647 | Santa Cruz Biotechnology | Sc-5275/MX-49.129.5 |
| 7D5 (Anti- Flavocytochromeb558) | FITC | MBL | D162-4/7D5 |
| CD55 | FITC | Diaclone | 853.371.010/MEM-118 |
| CD59 | FITC | Diaclone | 954.361.010/MEM43 |
| CD163 | FITC | ThermoFisher Scientific | MA5-17719/GHI-61 |
| **Adhesion markers** | | | |
| CD11b | FITC | BioRad | MCA551/ICRF44 |
| CD18 | FITC | Diaclone | 852.541.010/MEM48 |
| **Maturation markers** | | | |
| EMR3 | FITC | BioRad | MCA2476F/3D7 |
| CD16 | FITC | BD Pharmingen | 555406/3G8 |

Overview of all antibodies used for phenotyping neutrophils. Neutrophil markers were generally subdivided in groups of activation, adhesion and maturation based on literature to facilitate readability. Directly conjugated antibodies were used.

Abbreviations: CD, cluster of differentiation; FITC, fluorescein isothiocyanate; APC, allophycocyanin; AF, alexa fluor; CXCR, chemokine receptor; EGF-like module-containing mucin-like hormone receptor-like 3.

**Supplementary Table 2. Neutrophil adhesion and oxidative burst in healthy controls versus steady-state SCD patients.**

|  | **Steady state SCD**  **N=48** | **Matched controls**  **N=16** | **Non-matched controls**  **N=30** | ***p*** |
| --- | --- | --- | --- | --- |
| **Neutrophil adhesion** | | | | |
| Unstimulated | 18.2 [14.3-23.2] | 19.5 [13.3-25.0] | 12.3 [6.9-15.5] | **<.001^1^** |
| G-CSF | 18.5 [13.1-25.6] | 21.3 [13.3-25.4] | 12.5 [8.9-16.9] | **.002^1^** |
| DTT | 54.6 [48.5-57.6] | 53.0 [47.5-60.9] | 53.5 [49.2-56.7] | .972 |
| LBP/LPS | 31.0 [26.1-36.7] | 30.7 [25.9-35.9] | 26.4 [21.5-32.0] | **.010^2^** |
| Pam3Cys | 35.9 [27.5-42.2] | 36.9 [32.6-42.9] | 26.5 [18.8-33.2] | **<.001^1^** |
| PAF | 32.0 [29.0-37.5] | 32.9 [29.0-37.9] | 27.5 [22.6-30.8] | **.002^1^** |
| fMLP | 36.6 [27.7-44.9] | 36.8 [29.5-43.3] | 30.3 [24.6-36.9] | .131 |
| TNFa | 25.4 [19.6-32.7] | 26.7 [22.1-32.9] | 20.6 [17.8-26.6] | **.018^3^** |
| PMA | 47.4 [43.1-50.2] | 48.1 [44.1-52.7] | 46.9 [43.4-51.6] | .736 |
| **Neutrophil oxidative burst** | | | | |
| Unstimulated | 0.047 [0.042-0.055] | 0.049 [0.043-0.062] | 0.050 [0.041-0.059] | .648 |
| Zymosan | 0.40 [0.36-0.43] | 0.44 [0.40-0.50] | 0.42 [0.38-0.47] | **.029^4^** |
| STZ | 0.59 [0.52-0.63] | 0.63 [0.54-0.68] | 0.56 [0.51-0.66] | .244 |
| PMA | 1.22 [1.14-1.33] | 1.23 [1.13-1.31] | 1.24 [1.17-1.38] | .592 |
| fMLP | 0.60 [0.51-0.84] | 0.53 [0.31-0.83] | 0.72 [0.47-0.85] | .330 |
| PAF-fMLP | 1.39 [1.24-1.46] | 1.30 [1.10-1.43] | 1.41 [1.27-1.49] | .274 |

Results of neutrophil adhesion and oxidative burst assay (reactive oxygen species production by neutrophils) in SCD patients in steady state, ethnicity-matched healthy controls, and non-matched healthy controls.

* The *p* value represents the significance of the comparison between the three groups unless stated otherwise, a specification for significant *p* values is provided:

^1^ Significant difference between non-matched controls and matched controls, and between non-matched controls and steady-state SCD patients

^2^ Significant difference between non-matched controls and steady-state SCD patients

^3^ Significant difference between non-matched controls and matched controls

^4^ Significant difference between matched controls and steady-state SCD patients

All analyses were performed with subsequent correction for multiple testing with Dunn’s test.

Abbreviations: G-CSF, granulocyte colony-stimulating factor; DTT, dithiothreitol; LBP, lipopolysaccharide-binding protein; LPS, lipopolysaccharide; Pam3Cys, N-palmitoyl-S-(2,3-bis(palmitoyloxy)-(2RS)-propyl)-Cys-Ser-Lys4 hydrochloride; PAF, platelet-activation factor; fMLP, N-formyl-Met-Leu-Phe; TNF-α, tumor necrosis factor-α; PMA, phorbol myristate acetate; STZ, serum-opsonized zymosan.

**Supplementary Table 3. Neutrophil adhesion and oxidative burst in SCD patients in steady state and during VOC.**

|  | **Steady state**  **N=18** | **VOC**  **N=19** | ***p*** |
| --- | --- | --- | --- |
| **Neutrophil adhesion** | | | |
| Unstimulated | 18.9 [13.1-24.8] | 18.5 [13.2-31.3] | .170 |
| G-CSF | 18.5 [12.8-32.0] | 17.7 [13.2-35.7] | .647 |
| DTT | 55.4 [50.2-59.1] | 54.9 [51.3-59.1] | .811 |
| LBP/LPS | 29.5 [24.7-40.3] | 34.1 [25.7-46.1] | **.043** |
| Pam3Cys | 36.5 [28.6-43.9] | 40.5 [32.4-46.1] | .184 |
| PAF | 31.4 [28.9-37.0] | 34.6 [29.4-44.7] | .112 |
| fMLP | 38.0 [25.5-47.5] | 37.7 [32.5-51.0] | .420 |
| TNFa | 23.6 [19.2-34.6] | 27.5 [21.0-36.3] | .089 |
| PMA | 47.6 [42.1-53.1] | 48.0 [45.9-53.3] | .616 |
| **Neutrophil oxidative burst** | | | |
| Unstimulated | 0.047 [0.039-0.057] | 0.049 [0.044-0.079] | .084 |
| Zymosan | 0.40 [0.35-0.43] | 0.42 [0.35-0.45] | .066 |
| STZ | 0.57 [0.47-0.64] | 0.55 [0.48-0.65] | .368 |
| PMA | 1.19 [1.07-1.34] | 1.18 [0.95-1.43] | .845 |
| fMLP | 0.59 [0.51-0.67] | 0.50 [0.29-0.81] | .372 |
| PAF-fMLP | 1.32 [1.20-1.41] | 1.27 [1.09-1.49] | .913 |

Results of neutrophil adhesion and oxidative burst assay (reactive oxygen species production by neutrophils) in SCD patients in steady-state compared to SCD patients during vaso-occlusive crisis (paired data). Of one patient, no paired steady-state data was available.

Abbreviations: VOC, vaso-occlusive crisis; G-CSF, granulocyte colony-stimulating factor; DTT, dithiothreitol; LBP, lipopolysaccharide-binding protein; LPS, lipopolysaccharide; Pam3Cys, N-palmitoyl-S-(2,3-bis(palmitoyloxy)-(2RS)-propyl)-Cys-Ser-Lys4 hydrochloride; PAF, platelet-activation factor; fMLP, N-formyl-Met-Leu-Phe; TNF-α, tumor necrosis factor-α; PMA, phorbol myristate acetate; STZ, serum-opsonized zymosan.

**Supplementary Table 4. Neutrophil adhesion and oxidative burst in SCD patients before and during hydroxyurea treatment.**

|  | **Before HU**  **N=9** | **During HU**  **N=9** | ***p*** |
| --- | --- | --- | --- |
| **Neutrophil adhesion** | | | |
| Unstimulated | 14.3 [9.4-17.9] | 18.8 [14.5-24.1] | **.038** |
| G-CSF | 13.2 [9.8-19.5] | 17.9 [12.5-24.3] | .110 |
| DTT | 54.6 [47.9-56.5] | 52.8 [50.7-58.4] | .374 |
| LBP/LPS | 27.9 [24.1-32.9] | 30.6 [26.5-36.9] | .069 |
| Pam3Cys | 25.2 [22.3-37.5] | 39.6 [22.4-42.2] | .093 |
| PAF | 33.6 [25.9-39.4] | 34.4 [29.6-37.6] | .441 |
| fMLP | 30.7 [26.4-43.2] | 37.2 [31.0-42.4] | .173 |
| TNFa | 24.5 [20.4-26.5] | 26.1 [21.5-35.9] | .086 |
| PMA | 46.8 [42.9-49.4] | 50.8 [45.7-53.4] | .214 |
| **Neutrophil oxidative burst** | | | |
| Unstimulated | 0.043 [0.040-0.049] | 0.04 [0.031-0.050] | .128 |
| Zymosan | 0.42 [0.37-0.44] | 0.38 [0.33-0.43] | .208 |
| STZ | 0.60 [I0.53-0.66] | 0.54 [0.42-0.58] | .161 |
| PMA | 1.19 [1.15-1.25] | 1.10 [0.95-1.16] | .069 |
| fMLP | 0.56 [0.39-0.77] | 0.43 [0.35-0.47] | .161 |
| PAF-fMLP | 1.32 [1.19-1.49] | 1.14 [0.97-1.23] | **.036** |

Results of neutrophil adhesion and oxidative burst assay (reactive oxygen species production by neutrophils) in SCD patients before and during hydroxyurea treatment (paired data).

Abbreviations: HU, hydroxyurea; G-CSF, granulocyte colony-stimulating factor; DTT, dithiothreitol; LBP, lipopolysaccharide-binding protein; LPS, lipopolysaccharide; Pam3Cys, N-palmitoyl-S-(2,3-bis(palmitoyloxy)-(2RS)-propyl)-Cys-Ser-Lys4 hydrochloride; PAF, platelet-activation factor; fMLP, N-formyl-Met-Leu-Phe; TNF-α, tumor necrosis factor-α; PMA, phorbol myristate acetate; STZ, serum-opsonized zymosan

**Supplementary Table 5. Neutrophil adhesion and oxidative burst in SCD patients before and after HSCT.**

|  | **Before HSCT**  **N=7** | **After HSCT**  **N=7** | ***p*** |
| --- | --- | --- | --- |
| **Neutrophil adhesion** | | | |
| Unstimulated | 17.1 [13.9-26.2] | 16.0 [8.9-22.4] | .173 |
| G-CSF | 17.6 [14.6-25.8] | 17.3 [6.7-24.1] | .249 |
| DTT | 50.8 [45.6-57.9] | 53.4 [51.0-56.8] | .917 |
| LBP/LPS | 28.2 [23.2-35.0] | 25.1 [12.8-37.2] | .138 |
| Pam3Cys | 29.5 [23.0-42.1] | 25.5 [17.2-43.7] | .500 |
| PAF | 30.1 [27.1-38.3] | 35.75 [29.7-40.6] | .500 |
| fMLP | 33.2 [27.1-42.4] | 29.7 [21.7-37.3] | .080 |
| TNFa | 22.8 [17.6-32.4] | 25.1 [19.4-29.4] | .249 |
| PMA | 44.3 [41.4-49.2] | 49.29 [46.38-50.17] | .345 |
| **Neutrophil oxidative burst** | | | |
| Unstimulated | 0.044 [0.040-0.057] | 0.040 [0.039-0.043] | .141 |
| Zymosan | 0.38 [0.35-0.42] | 0.34 [0.32-0.40] | .249 |
| STZ | 0.55 [0.49-0.58] | 0.50 [0.45-0.58] | .463 |
| PMA | 1.19 [1.15-1.24] | 1.10 [1.01-1.20] | .173 |
| fMLP | 0.80 [0.49-0.90] | 0.47 [0.40-0.77] | .249 |
| PAF-fMLP | 1.36 [1.24-1.46] | 0.96 [0.92-1.24] | .116 |

Results of neutrophil adhesion and oxidative burst assay (reactive oxygen species production by neutrophils) in SCD patients before and after hematopoietic stem cell transplantation (paired data).

Abbreviations: HSCT, hematopoietic stem cell transplantation SCT, G-CSF, granulocyte colony-stimulating factor; DTT, dithiothreitol; LBP, lipopolysaccharide-binding protein; LPS, lipopolysaccharide; Pam3Cys; N-palmitoyl-S-(2;3-bis(palmitoyloxy)-(2RS)-propyl)-Cys-Ser-Lys4 hydrochloride; PAF, platelet-activation factor; fMLP, N-formyl-Met-Leu-Phe; TNF- α, tumor necrosis factor-α; PMA, phorbol myristate acetate; STZ, serum-opsonized zymosan.
